# Neurosurgical management for chronic and end‐of‐life pain in children: A systematic review

**DOI:** 10.1111/papr.70034

**Published:** 2025-04-10

**Authors:** Sunny Abdelmageed, Nicole Villalba, Gloria Bae, James M. Mossner, Siegfried J. Adelhoefer, Kannan Aravagiri, Ravi D. Shah, Jeffrey S. Raskin

**Affiliations:** ^1^ Division of Pediatric Neurosurgery Ann and Robert H. Lurie Children's Hospital of Chicago Chicago Illinois USA; ^2^ Department of Neurosurgery Northwestern University Feinberg School of Medicine Chicago Illinois USA; ^3^ Chicago Medical School Rosalind Franklin University of Medicine and Science North Chicago Illinois USA; ^4^ Faculty of Medicine Charité–Universitätsmedizin Berlin Berlin Germany; ^5^ Division of Pediatric Anesthesiology Ann and Robert H. Lurie Children's Hospital of Chicago Chicago Illinois USA

**Keywords:** analgesia, intrathecal, neurosurgical procedures, pediatric pain, spinal cord, stimulation

## Abstract

**Introduction:**

Chronic and end‐of‐life pain in children is underreported and undermanaged. Current guidelines for pediatric chronic pain include medical and interventional modalities; however, the inclusion of neurosurgical treatments is uncommon and inconsistent. This systematic review presents the literature, and we provide recommendations for the role of neurosurgical procedures in treating chronic and end‐of‐life pain in children.

**Methods:**

A systematic review was conducted in accordance with the Preferred Reporting Items for Systematic Review and Meta‐analyses (PRISMA) guidelines using three databases: PubMed, Embase, and Scopus. We included 40 studies presenting neurosurgical procedures for the treatment of chronic and end‐of‐life pain in children.

**Results:**

Thirty‐one (77.5%) manuscripts focused on the treatment of neuropathic pain, five (12.5%) focused on nociceptive pain, and four (10%) treated mixed pain conditions. The most common neurosurgical procedure was intrathecal opioid therapy via pump placement (29.3%), followed by spinal cord stimulation (26.8%). Neuropathic pain syndromes were primarily treated with neurostimulation (58%), demonstrating good efficacy. Ablative procedures (40%) were most effective for nociceptive pain syndromes. Both chordotomy and intrathecal pumps provided subjective pain relief for mixed pain syndromes. The quantification of procedural efficacy, including pain outcomes and grading scales, varied significantly across studies.

**Conclusion:**

Neurosurgical treatments for chronic pediatric pain are safe, although broad efficacy cannot be determined due to sparse literature and inadequately quantified pain responses. Guidelines for escalating chronic and end‐of‐life pain management in pediatric patients should be updated to include neurosurgical treatments and appropriate outcome scales. Focused research on appropriate patients, available neurosurgical therapies, and pediatric outcomes is warranted.

## INTRODUCTION

Chronic pain, defined as persistent or recurring pain lasting longer than 3 months, has high morbidity and long‐lasting quality of life impact.^1^ In pediatric populations, chronic pain of any type is estimated to impact between 11% and 38% of individuals, with approximately 5% of those with chronic pain experiencing significant impacts in daily functioning.^2^ Chronic pain has noticeable impacts on a child's future development and can lead to education delays or long‐lasting psychological harm.^2^, ^3^

Pediatric chronic pain requires interdisciplinary pain management by a team of healthcare professionals from various disciplines. Treatment approaches include physical therapy, occupational therapy, psychological support, and medication.^4^ Initially, conservative medical management focuses on nonopioid medications, often evolving to opioid therapies of increasing strength. When these conservative measures fail, interventional procedures are often introduced. Common regional and neuraxial injections are considerably less invasive than neurosurgical procedures.^5^, ^6^ These techniques demonstrate a safety and efficacy profile for pediatric chronic pain similar to those in adults.^7^ However, neurosurgical interventions in the pediatric population are less well studied and are not currently included in chronic or end‐of‐life pain management guidelines.^8^

The neurosurgical treatment of pain can be categorized into three groups: neurostimulation, ablative techniques, and targeted drug delivery through intrathecal pumps.^5^ These neurosurgical modalities have been shown to vary in efficacy for different types of pain. Understanding the fundamental primary pain generators for chronic and end‐of‐life pain is essential to optimize neurosurgical therapy. Pain is often defined by its pathophysiology, and common types include nociceptive (ie, originating from nociceptors in the musculoskeletal system), neuropathic (ie, originating from the central nervous system), and mixed. Nociplastic pain is an alteration of perception pain syndrome (eg, fibromyalgia) and is not covered in this review.^9^

Comprehensive reviews of the neurosurgical treatment of pain in adults stipulate indications and outcomes across procedures and pain syndromes; however, this is entirely lacking for children.^5^ We systematically review the existing literature regarding the neurosurgical treatment of chronic and end‐of‐life pain in pediatric populations and provide recommendations for neurosurgical procedures in practice.

## METHODS

### Study selection

A systematic review was conducted in accordance with the Preferred Reviews and Meta‐analyses (PRISMA) 2020 guidelines to determine the role of neurosurgical procedures in the treatment of chronic pain in pediatric patients.^10^ Keywords associated with chronic pain, pediatric patients, and neurosurgical procedures were searched on November 27, 2023, through PubMed MEDLINE (National Library of Medicine), Embase (Elsevier), and Scopus (Elsevier). A full list of search terms is listed in Table S1. No date, language, or article type restrictions were applied. This protocol was prospectively registered on PROSPERO.^11^

All publications from the initial search were imported into EndNote (Clarivate Analytics, Philadelphia, PA), and duplicates were excluded. The deduplicated articles were screened for relevance by title and abstract by two authors (SA and GB). Articles progressing to full‐text review were screened for final inclusion according to the following predetermined inclusion criteria: (1) published in or translated into the English language, (2) available full text, (3) pediatric population (<25 years of age) with chronic pain, (4) neurosurgical therapy for the primary purpose of pain control, and (5) provided outcomes of neurosurgical procedures. The outcomes of treatment included complication rates and efficacy determined by improvement in pain scales. Articles were screened independently by two reviewers (SA and GB) through Rayyan (https://rayyan.qcri.org/). All disagreements were discussed and resolved by the reviewers.

### Data extraction

Data from included studies were extracted independently and cross‐checked for accuracy by authors (SA and GB). Article information including bibliographic data, study design, number of patients, condition, type of pain, procedure, and outcomes—efficacy and complications—were reviewed.

### Type of pain

Studies were grouped into three categories based on the type of pain (neuropathic, nociceptive, or mixed). The type of pain was determined in the following tiered system: (1) reported within the study manuscript, (2) pain condition with known etiology, and (3) classified by authors through standard definitions of pain types. Any manuscript needing to be classified by authors was reviewed by a senior author (JSR) for accuracy.

### Pain outcomes

The primary outcome was efficacy of the neurosurgical procedure to treat pain. Efficacy was determined for studies that reported numerical pain scores pre‐ and postoperatively. Treatment was determined effective if there was a ≥50% reduction in the numeric pain score and ineffective if there was a <50% reduction. Studies that did not report a numeric pain score or had missing data were classified as “not quantified.” Subjective data on the effect on pain control were collected, but these data were not used to determine efficacy. Secondary outcomes included both intraoperative and postoperative complications. Descriptive analysis was performed in Excel.

### Risk of bias and quality evaluation

Critical appraisal of all included studies was conducted by assessing the risk of bias using the Risk Of Bias In Non‐Randomized Studies—of Interventions (ROBINS‐I) tool and quality using the Grading of Study Design Quality framework as described in another study.^12^, ^13^ Two reviewers (SA, GB) assessed the risk of bias and quality of each study independently, and disagreements were resolved by a third reviewer (NV).

## RESULTS

A total of 3492 articles were identified: 990 from PubMed, 915 from Scopus, and 1587 from Embase. 858 duplicate articles were removed, and 2634 articles were screened by title and abstract. After initial title and abstract screening, 139 full‐text articles were assessed for eligibility and 40 articles were found to be eligible (Figure S1).^14^, ^15^, ^16^, ^17^, ^18^, ^19^, ^20^, ^21^, ^22^, ^23^, ^24^, ^25^, ^26^, ^27^, ^28^, ^29^, ^30^, ^31^, ^32^, ^33^, ^34^, ^35^, ^36^, ^37^, ^38^, ^39^, ^40^, ^41^, ^42^, ^43^, ^44^, ^45^, ^46^, ^47^, ^48^, ^49^, ^50^, ^51^, ^52^, ^53^ Study characteristics are reported in Table 1. The study periods ranged from 1992 to 2023. The majority of studies were case reports (72.5%) followed by case series (12.5%), retrospective cohorts (10%), prospective cohorts (2.5%), and randomized clinical trials (2.5%). The number of patients in each study ranged from 1 to 88. In total, there were 281 pediatric participants with chronic pain who received neurosurgical treatment. The overall risk of bias in this study was serious, and the overall quality of evidence was low due to the prevalence of case reports and lack of randomized control trials included.

**TABLE 1:** Study characteristics.

Study	Country	Study design (*n*)	Follow‐up period	Pain diagnosis	Procedure	Pain outcomes	Complications	Time to surgery decision	Quality/risk of bias
Reddy et al^14^	USA	Case report (1)	10 days	Intractable cancer pain	Percutaneous cordotomy	Pain relief, functional status	None	Unspecified	E/Serious
Steel et al^15^	UK	Case report (2)	12 weeks, 36 months	Intractable cancer pain	Open thoracic anterolateral cordotomy	Pain relief, functional status, medication use	None	2 months	E/Serious
Kanpolat et al^16^	Turkey	Case report (1)	60 months	CRPS‐I	DREZotomy	VAS score, functional status	Transient ipsilateral lower extremity ataxia (2 months)	19 months	E/Serious
Iglesias et al., 2022^17^	Argentina	Case series (3)	Unspecified, 4 months, unspecified	Intractable cancer pain	Lumbosacral DREZotomy	VAS score, quality of life	None	Unspecified, 4 months, 19 months	E/Serious
D'Angelo et al^18^	USA	Case report (1)	1 year	Erythromelalgia	Intrathecal pump	Pain relief	None	2 weeks	E/Serious
Collins et al^19^	USA	Retrospective study (11)	Unspecified	Intractable cancer pain	Intrathecal pump	Pain relief	Catheter occlusion by tumor causing tachyphylaxis	2 weeks‐6 months	B/Moderate
Galloway et al^20^	USA	Case report (1)	5 months	Intractable cancer pain	Intrathecal pump	VAS score, functional status, quality of life	None	Unspecified	E/Serious
Aram et al^21^	USA	Retrospective study (25)	4–240 days	Intractable cancer pain, abdominal surgery, trauma	Intrathecal pump	Pain relief, medication use	Cellulitis at catheter exit site, accidental dislodgement	Unspecified	B/Serious
Stanton‐Hicks & Kapural^22^	USA	Case report (1)	9 months	CRPS‐I	Intrathecal pump	VAS score, functional status	None	2 months	E/Serious
Farid & Heiner^23^	USA	Case report (1)	2 months	CRPS	Intrathecal pump	Pain relief, functional status	None	7 weeks	E/Serious
Kajiume et al^24^	Japan	Case report (1)	109 days	Intractable cancer pain	Intrathecal pump^a^	Pain relief, quality of life	None	57 days	E/Serious
Moens et al^25^	Belgium	Case report (1)	3 months	Pain due to arachnoiditis ossificans	Intrathecal pump	Pain relief	Transient loss of appetite, metallic taste	Unspecified	E/Serious
Bengali et al^26^	USA	Case report (1)	5 months	Intractable cancer Pain	Intrathecal pump	Pain relief, quality of life	None	Unspecified	E/Serious
Abolhasan Gharehdaghi et al^27^	Iran	Randomized clinical trial (88)	14 days	Intractable cancer pain	Intrathecal pump^a^	VRS score, VAS score, DN4 score	Nausea, vomiting	Unspecified	A/Moderate
Tubic^28^	USA	Case report (1)	12 weeks	CRPS‐I	Intrathecal pump	NRS score, functional status	Transient itching on stomach, nausea, stomach irritation	2 months	E/Serious
Bentley et al^29^	USA	Case report (1)	3 months	Intractable cancer pain	Intrathecal pump + midline myelotomy	VAS score	None	Unspecified	E/Serious
Rodriguez‐Lopez et al^30^	Spain	Case series (6)	Unspecified	CRPS	Intrathecal pump + spinal cord stimulation	VAS score, functional status, medication use, pain relief	None	5 weeks	E/Serious
Kim et al^31^	USA	Case series (14)	22 months +/− 18 months	Intractable pain	Intrathecal pump, spinal cord stimulation	VNS score, functional status, medication use	Seroma, post‐dural puncture headache, pump erosion through skin, vomiting	6–58 months	E/Serious
Ivanishvili et al^32^	UK	Case report (1)	5 months	Intractable cancer pain	Stereotactic mesencephalotomy	VAS score, medication use	None	6 months	E/Serious
Kato et al^33^	Japan	Case report (1)	3 months	Trigeminal neuralgia	Rhizotomy	Pain relief	Transient Mild sensory disturbance (tinnitus, hearing loss, left nystagmus)	8 months	E/Serious
Perides et al^34^	UK	Retrospective study (63)	12.4 months +/1.2 months	Dystonic pain	Deep brain stimulation	NRS score, PPP, medication use	None	Unspecified	B/Serious
Mol & Roumen^35^	Netherlands	Case report (1)	12 months	Anterior cutaneous nerve entrapment syndrome	Dorsal root ganglion stimulation	NRS score	Severe pain at battery site improved with relocation	Unspecified	E/Serious
Pinckard‐Dover et al^36^	USA	Case report (1)	30 months	CRPS‐I	Dorsal root ganglion stimulation	Pain relief, functional status	None	2 years	E/Serious
Graca et al^37^	USA	Case series (5)	3–48 months	CRPS‐I	Dorsal root ganglion stimulation	NRS score, functional status	None	1–5 years	E/Serious
Vles et al^38^	Netherlands	Prospective study (17)	6–9 months	Spastic pain	Radiofrequency lesion of dorsal root ganglia	VAS score	Transient increase in pain	Unspecified	B/Moderate
Apiliogullari et al^39^	Turkey	Case report (1)	6 months	CRPS‐I	Pulsed radiofrequency to dorsal root ganglia	VAS score	None	1 month	E/Serious
Delavallee et al^40^	Belgium	Case report (1)	15 months	Trigeminal neuralgia	Motor cortex stimulation	Pain relief, medication use	Superficial wound infection	Unspecified	E/Serious
Stubberud et al^41^	Norway	Case report (1)	3 years	Headache	Occipital nerve stimulation	Pain relief	None	5 years	E/Serious
Borius & Valade^42^	France	Case report (1)	12 months	Headache	Occipital nerve stimulation	Pain relief, quality of life	None	6 months	E/Serious
Olsson et al^43^	Sweden	Retrospective case study (7)	1–3 years	CRPS‐I	Spinal cord stimulation	Pain relief, functional status	Subcutaneous infection	Unspecified	B/Serious
Patel et al^44^	USA	Case report (1)	2 years	Erythromelalgia	Spinal cord stimulation	NRS score, functional status	None	Unspecified	E/Serious
Dones et al^45^	Italy	Case report (1)	1 year	Lymphangioma	Spinal cord stimulation	VAS score, functional status	None	4 years	E/Serious
Tyagi et al^46^	USA	Case report (1)	10 months	Recurrent tethered cord syndrome	Spinal cord stimulation	VAS score, functional status	None	Unspecified	E/Serious
Fan et al^47^	China	Case report (1)	12 months	Erythromelalgia	Spinal cord stimulation	VAS score, medication use	None	Unspecified	E/Serious
Hale & Cheng^48^	USA	Case report (1)	3 years	Neurogenic thoracic outlet syndrome	Spinal cord stimulation	Pain relief, functional status, medication use	None	1 year	E/Serious
Bakr et al^49^	USA	Case series (12)	12 months	CRPS, spinal cord injury, hyperpathia, lumbago, urogenic pain, neuralgia	Spinal cord stimulation	VAS score, medication use, functional status	Transient intraoperative CSF leak	1–6 years	E/Serious
Schatmeyer et al^50^	USA	Case report (1)	6 months	CRPS	Spinal cord stimulation	Pain relief, quality of life	None	Unspecified	E/Serious
Toriya et al^51^	Russia	Case report (1)	6 months	Erythromelalgia	Spinal cord stimulation	VAS score, functional status	None	Unspecified	E/Serious
Zuo et al^52^	China	Case report (1)	2 months	Erythromelalgia	Spinal cord stimulation	VAS score, functional status, medication use	None	1 year	E/Serious
Delye et al^53^	Belgium	Case report (1)	7 weeks	Erythromelalgia	Deep brain stimulation	Pain relief, medication use	None	Unspecified	E/Serious

Abbreviations: CSF, cerebrospinal fluid; CRPS, chronic regional pain syndrome; CRPS‐I, chronic regional pain syndrome type 1; DN4, Douleur Neuropathique 4; DREZ, dorsal root entry zone; NRS, Numeric Rating Scale; PPP, Pediatric Pain Profile; VAS, Visual Analogue Scale; VNS, Verbal Numeric Scale; VRS, Verbal Rating Scale; UK, United Kingdom; USA, United States of America.

^a^ Intrathecal injection.

### Pain diagnoses and neurosurgical procedures

**FIGURE 1:**
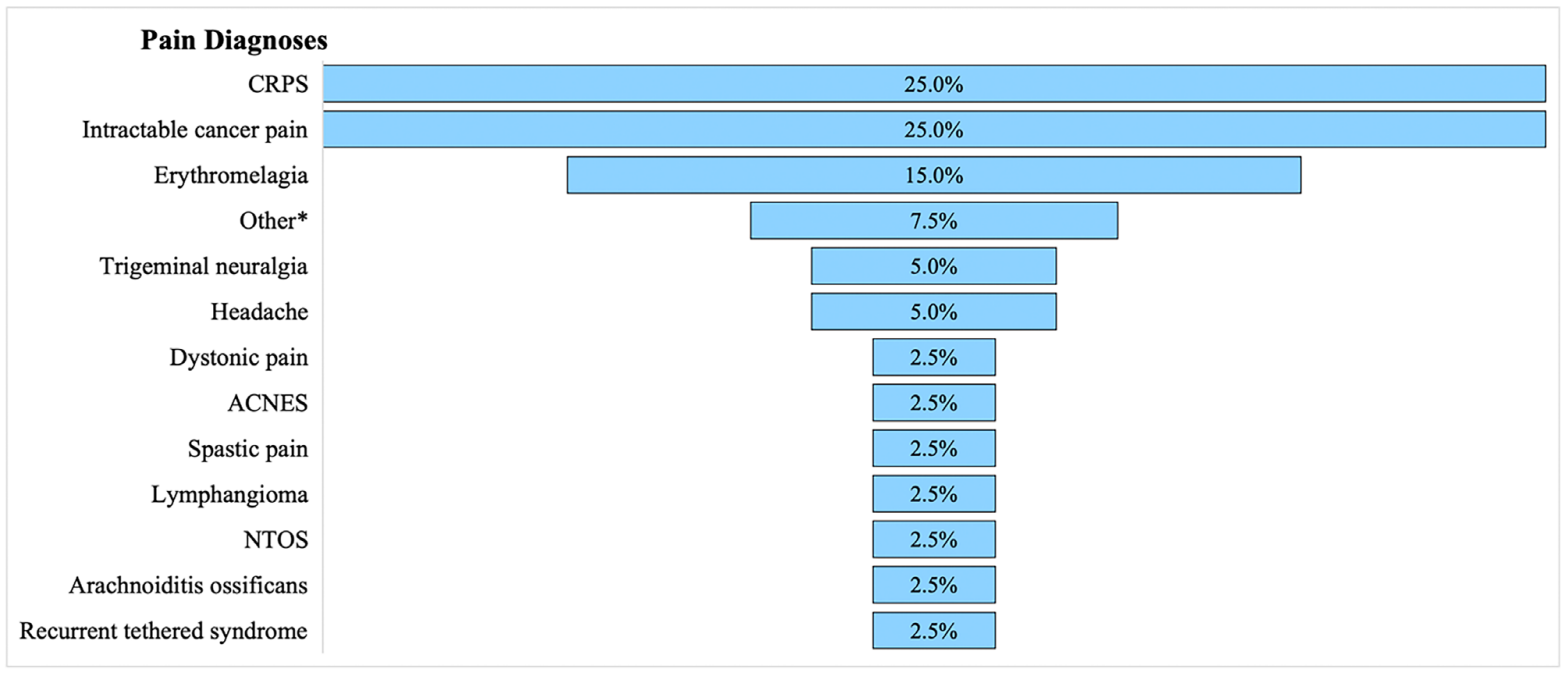
Funnel plot of pain diagnoses reported in the included studies, from most commonly (top) to least commonly (bottom) reported. ACNES, anterior cutaneous nerve entrapment syndrome; CRPS, chronic regional pain syndrome; NTOS, neurogenic thoracic outlet syndrome. *other includes studies (Aram 2001, Bakr 2022, and Kim 2018) including patients with multiple etiologies of chronic pain these include cancer/metastatic disease, hematologic conditions, severe trauma causing lasting injury, chronic low back pain, spinal cord injury, musculoskeletal conditions, cerebral palsy.

**FIGURE 2:**
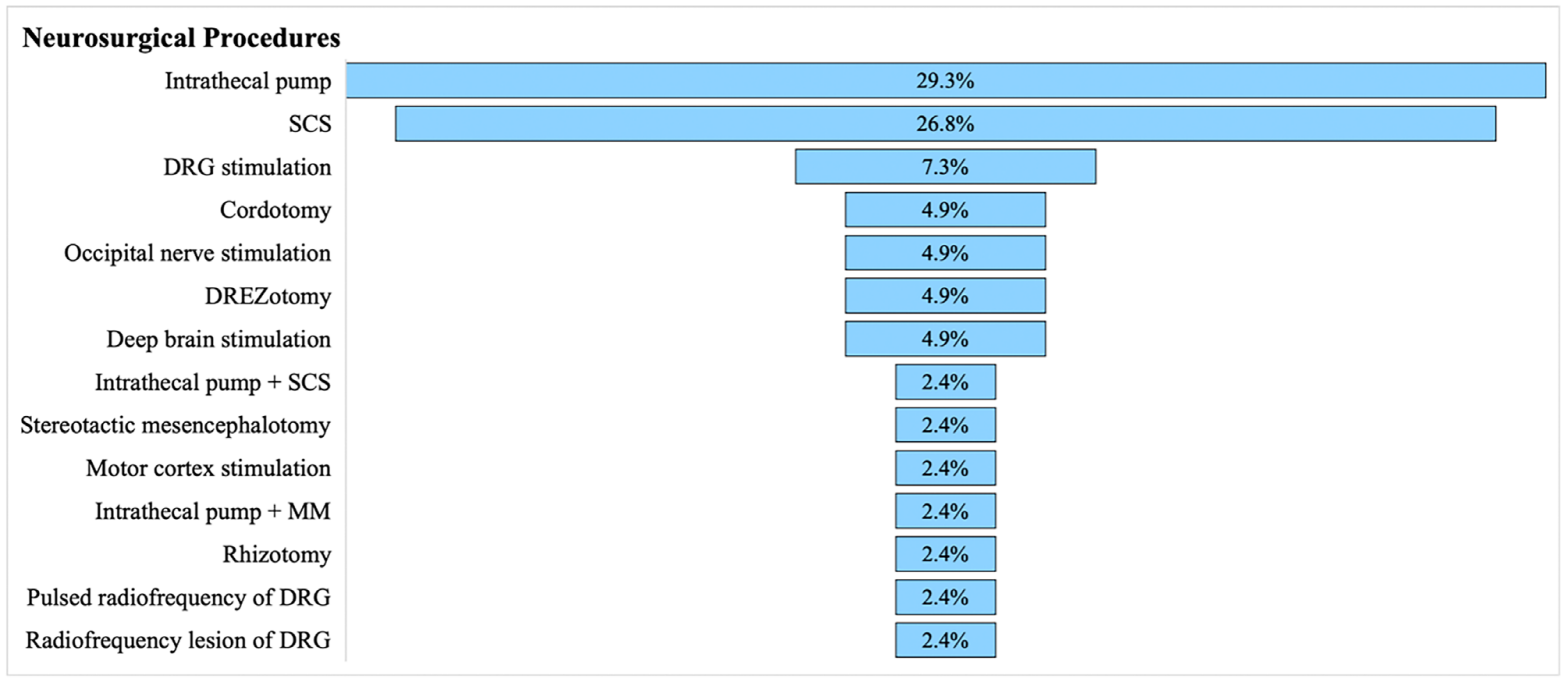
Funnel plot of neurosurgical procedures for pain reported in the included studies, from most commonly (top) to least commonly (bottom) reported. DREZ, dorsal root entry zone; DRG, dorsal root ganglion; MM, midline myelotomy.

Of the 40 studies, 31 (77.5%) reported a neuropathic type of pain,^16^, ^17^, ^18^, ^19^, ^20^, ^22^, ^23^, ^24^, ^26^, ^27^, ^28^, ^30^, ^31^, ^33^, ^35^, ^36^, ^37^, ^39^, ^40^, ^42^, ^43^, ^44^, ^45^, ^46^, ^47^, ^48^, ^49^, ^50^, ^51^, ^52^, ^53^ 5 (12.5%) reported a nociceptive pain,^25^, ^32^, ^34^, ^38^, ^41^ and 4 (10%) reported a mixed pain (Table 2).^14^, ^15^, ^21^, ^29^ The most common diagnoses were intractable cancer pain and complex regional pain syndrome (CRPS). Both diagnoses were found in 10 (25%) articles each (Figure 1).^14^, ^15^, ^16^, ^17^, ^19^, ^20^, ^22^, ^23^, ^24^, ^26^, ^27^, ^28^, ^29^, ^30^, ^32^, ^36^, ^37^, ^39^, ^43^, ^50^ Of the 41 neurosurgical procedures for pain reported in all studies, the intrathecal pump was the most commonly used (29.3%),^18^, ^19^, ^20^, ^21^, ^22^, ^23^, ^24^, ^25^, ^26^, ^27^, ^28^, ^31^ followed by spinal cord stimulation (SCS) (26.8%),^31^, ^43^, ^44^, ^45^, ^46^, ^47^, ^48^, ^49^, ^50^, ^51^, ^52^ dorsal root ganglion (DRG) stimulation (7.3%),^35^, ^36^, ^37^ cite cordotomy (4.9%),^14^, ^15^ occipital nerve stimulation (ONS) (4.9%),^41^, ^42^ lesioning of the dorsal root entry zone (DREZotomy) (4.9%),^16^, ^17^ deep brain stimulation (DBS) (4.9%),^34^, ^53^ and other procedures (intrathecal pump + SCS, mesencephalotomy, motor cortex stimulation (MCS), intrathecal pump + midline myelotomy, rhizotomy, pulsed radiofrequency of DRG, and radiofrequency lesion of DRG) each at 2.4% (Figure 2).^29^, ^30^, ^32^, ^33^, ^38^, ^39^, ^40^

**TABLE 2:** Pain diagnoses reported in studies.

Neuropathic pain (*n* = 31)	Nociceptive pain (*n* = 5)	Mixed pain (*n* = 4)
CRPS (*n* = 10)	Arachnoiditis ossificans (*n* = 1)	Intractable cancer pain (*n* = 3)
Intractable cancer pain (*n* = 6)	Intractable cancer pain (*n* = 1)	Other^a^ (*n* = 1)
Erythromelalgia (*n* = 6)	Dystonic pain (*n* = 1)	
Trigeminal neuralgia (*n* = 2)	Spastic pain (*n* = 1)	
Other^a^ (*n* = 2)	Headache (*n* = 1)	
Anterior cutaneous nerve entrapment syndrome (*n* = 1)		
Headache (*n* = 1)		
Lymphangioma (*n* = 1)		
Neurogenic thoracic outlet syndrome (*n* = 1)		
Recurrent tethered cord syndrome (*n* = 1)		

Abbreviations: CRPS, chronic regional pain syndrome.

^a^ Heterogeneous etiologies of pain.

### Pain outcomes

Reported pain outcomes across studies were heterogeneous, with both qualitative and quantitative measures used (Table 3). The most common methods for reporting pain outcomes were qualitative measures, including pain relief (47.5%) and functional status (47.5%). Six quantitative assessments were used, with the Visual Analogue Scale (VAS) being the most frequent (40.0%), followed by the Numeric Rating Scale (NRS) (12.5%), the Karnofsky Performance Scale (5.0%), the Verbal Rating Scale (VRS) (2.5%), the Verbal Numeric Scale (VNS) (2.5%), and Douleur Neuropathique (DN4) (2.5%).

**TABLE 3:** Pain outcomes reported in studies.

Pain outcome	*N* (%)
Visual Analogue Scale (VAS)	16 (40.0%)
Numeric Rating Scale (NRS)	5 (12.5%)
Verbal Rating Scale (VRS)	1 (2.5%)
Verbal Numeric Scale (VNS)	1 (2.5%)
Douleur Neuropathique 4 (DN4)	1 (2.5%)
Karnofsky Performance Scale	2 (5.0%)
Pain relief	19 (47.5%)
Functional status (mobility, activity, strength)	19 (47.5%)
Quality of life	6 (15.0%)
Pain medication use	12 (30.0%)
Mood (depression, anxiety)	4 (10.0%)

**FIGURE 3:**
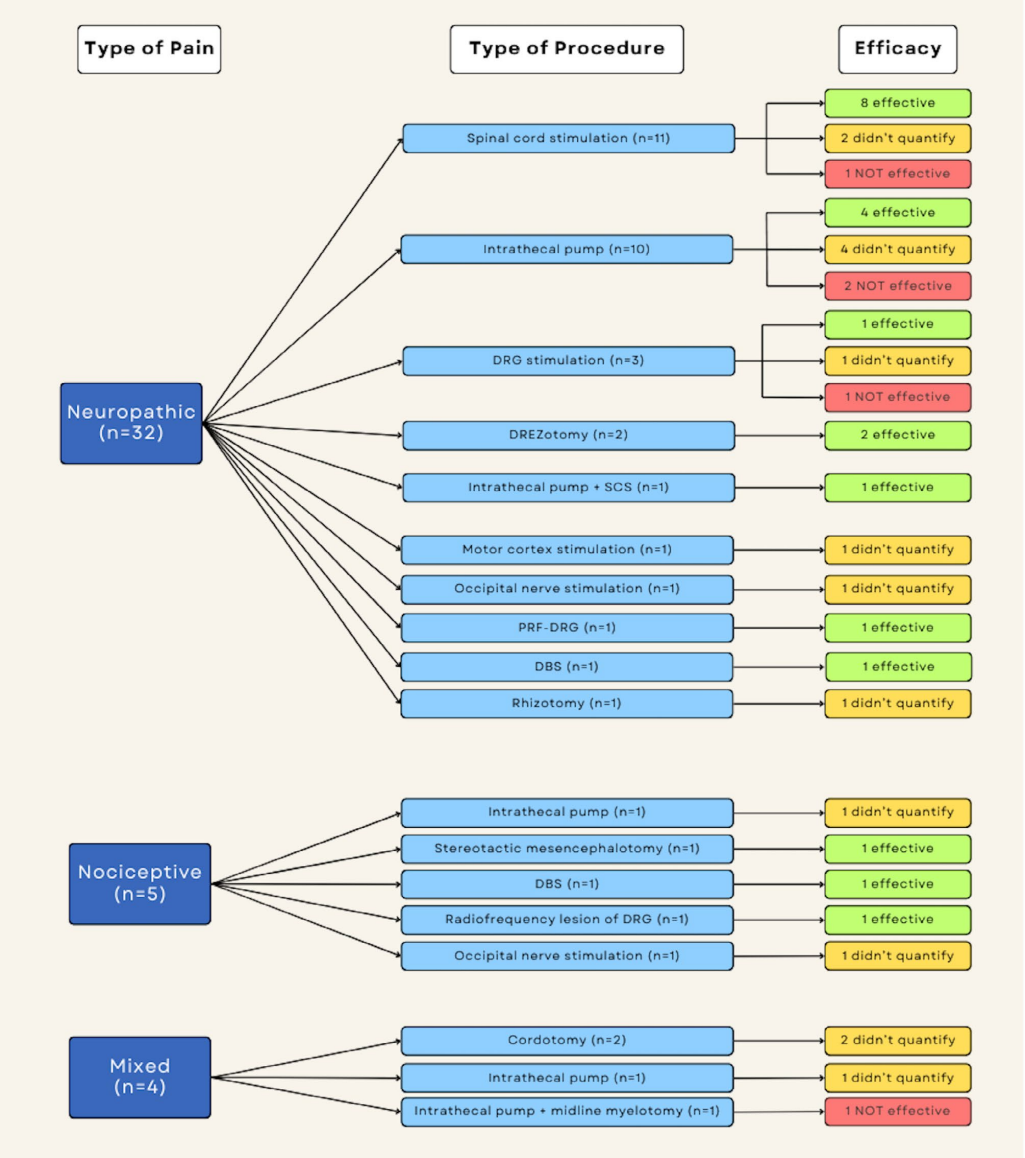
Efficacy of neurosurgical procedures reported in the included studies. The first column (dark blue, left) indicates the type of pain (neuropathic, nociceptive, or mixed) and the number of neurosurgical procedures by study employed. The middle column (light blue, middle) reports the type of procedure. The last column (right) reports the efficacy, with effective indicated in green, not quantified in yellow, and not effective in red. Efficacy was determined using only quantitative measures of pain control, defining effectiveness as a ≥50% reduction in the numeric pain score. DBS = deep brain stimulation; DREZ, dorsal root entry zone; PRF, pulsed radiofrequency; SCS, spinal cord stimulation.

The efficacy of neurosurgical pain treatments is reported in Figure 3. Neuropathic pain was treated with 32 procedures across 31 studies. SCS was the most common neurosurgical procedure reported in 35.5% (11/31) of studies.^31^, ^43^, ^44^, ^45^, ^46^, ^47^, ^48^, ^49^, ^50^, ^51^, ^52^ Of these studies, 8 reported effective treatment while 1 did not report improved results (Table 4).^31^, ^44^, ^45^, ^46^, ^47^, ^48^, ^49^, ^51^, ^52^ The remaining 2 studies reported significant improvement in pain relief and quality of life but did not provide numeric scores for a measurable outcome.^43^, ^50^ Other forms of treatment for neuropathic pain that were determined to be effective included intrathecal pump (*n* = 4),^20^, ^23^, ^27^, ^28^ DREZotomy (*n* = 2),^16^, ^17^ DRG stimulation (*n* = 1),^35^ intrathecal pump + SCS (*n* = 1),^30^ DBS (*n* = 1),^53^ and pulsed radiofrequency of DRG (*n* = 1) (Table 4).^39^ There were 10 studies that did not provide numeric pain scores but reported overall improvement in quality of life and pain relief after treatment of neuropathic pain, including intrathecal pump (*n* = 4),^18^, ^19^, ^24^, ^26^ SCS (*n* = 2),^43^, ^50^ DRG stimulation (*n* = 1),^36^ MCS (*n* = 1),^40^ ONS (*n* = 1),^42^ and rhizotomy (*n* = 1).^33^ There were 4 studies that reported ineffective treatment of chronic neuropathic pain, including intrathecal pump (*n* = 2),^22^, ^31^ SCS (*n* = 1),^31^ and DRG stimulation (*n* = 1) (Table 4).^37^

**TABLE 4:** Preoperative and postoperative pain scores.

Intrathecal pump					
Neuropathic pain					
Study	Procedure	Assessment	Preoperative Pain Score	Postoperative Pain Score	Effective?
Galloway et al^20^	Intrathecal pump	VAS	8	0–3	Yes
Stanton‐Hicks & Kapural^22^	Intrathecal pump	VAS	5	4	No
Farid & Heiner^23^	Intrathecal pump	NRS	10	0–2	Yes
Abolhasan Gharehdaghi et al^27^	Intrathecal pump	VAS	7 +/−0.25	1 +/−0.12	Yes
Kim et al^31^	Intrathecal pump	VNS	7.9	5.9	No
Tubic^28^	Intrathecal pump	NRS	8	0	Yes
Ablative techniques					
Neuropathic pain					
Kanpolat et al^16^	DREZotomy	VAS	9	0	Yes
Iglesias et al., 2022^17^	Lumbosacral DREZotomy	VAS	10	0.67	Yes
Apiliogullari et al^39^	Pulsed radiofrequency to dorsal root ganglion	VAS^a^	100	0	Yes
Nociceptive pain					
Vles et al^38^	Radiofrequency lesion of dorsal root ganglion	VAS	8.39	4.17	Yes
Ivanishvili et al^32^	Stereotactic mesencephalotomy	VAS	10	0	Yes
Neuromodulation					
Neuropathic pain					
Patel et al^44^	Spinal cord stimulation	NRS	10	0	Yes
Dones et al^45^	Spinal cord stimulation	VAS	9	1	Yes
Tyagi et al^46^	Spinal cord stimulation	VAS	8	1	Yes
Kim et al^31^	Spinal cord stimulation	VNS	7.5	3.8	No
Fan et al^47^	Spinal cord stimulation	VAS	8	0	Yes
Hale & Cheng^48^	Spinal cord stimulation	NRS	6	0–2	Yes
Bakr et al^49^	Spinal cord stimulation	VAS	9.2	2.9	Yes
Toriya et al^51^	Spinal cord stimulation	VAS	8	2–4	Yes
Zuo et al^52^	Spinal cord stimulation	VAS	6	2–3	Yes
Mol & Roumen^35^	Dorsal root ganglion stimulation	NRS	8	3	Yes
Graca et al^37^	Dorsal root ganglion stimulation	NRS	9.4	5.2	No
Nociceptive pain					
Perides et al^34^	Deep brain stimulation	NRS	7.3	2.3	Yes, *p* < 0.001
Mixed procedures					
Neuropathic pain					
Rodriguez‐Lopez et al^30^	Intrathecal pump + spinal cord stimulation	VAS	7.7	0.3	Yes
Mixed pain					
Bentley et al^29^	Intrathecal pump + midline myelotomy	VAS	9	7	No

Abbreviations: DREZ, dorsal root entry zone; NRS, Numeric Rating Scale; VAS, Visual Analogue Scale; VNS, Verbal Numeric Scale.

^a^ 100 mm VAS.

Nociceptive pain was treated with 5 different neurosurgical procedures across 5 studies.^25^, ^32^, ^34^, ^38^, ^41^ Two studies, one using an intrathecal opioid pump and the other using ONS, reported subjective pain relief but did not provide numeric pain scores.^25^, ^41^ The remaining 3 studies provided pain scores and were determined to be effective in their treatment of chronic pain with stereotactic mesencephalotomy, DBS, and radiofrequency lesion of the DRG (Table 4).^32^, ^34^, ^38^

Lastly, in the treatment of mixed pain, 2 studies reporting on cordotomy and 1 study using an intrathecal opioid pump reported subjective pain relief but did not quantify pain using a numeric score.^14^, ^15^, ^21^ In one study, combination therapy with an intrathecal opioid pump and midline myelotomy was determined to be not effective in the treatment of chronic pain (Table 4).^29^

### Complications

Overall, complications were minimal; see Table 1. The most common complication of ablative techniques was sensory complications. Difficulty with the pump or catheter system or medication side effects was the most common complication of intrathecal pumps. Infection was the most common complication of neurostimulation.

## DISCUSSION

Chronic pain is undertreated and underreported in pediatric populations.^3^ Chronic pain reduces quality of life and can affect the developmental trajectory in children.^2^, ^3^ Treatment protocols for chronic pain typically evolve from non‐interventional medication regimens and physical therapy to regional and neuraxial injections and finally to neurosurgical procedures. Studying pain sensation in children can differ from that in adults, and this suggests that chronic pain in children should be addressed with even more vigor.^54^ Neurosurgical pain procedures in children are less widely applied with limited literature and absent guidelines. Contributors to a lack of evidence and application of neurosurgical pain procedures in children include ethical considerations, a lack of FDA‐approved indications, adult hardware, and limited infrastructure in institutions dedicated to treating children. There is also a lack of reliable, standardized assessments of pre‐ and postoperative pain in children. A recent review discussing interventional procedures for chronic pain in children provides a comprehensive overview of regional, and neuraxial techniques yet exclude neurosurgical therapies apart from one case report of SCS.^7^ To our knowledge, this is the first comprehensive review of neurosurgical treatments for chronic pain in children.

Pain can be classified into three main categories: neuropathic, nociceptive, and mixed.^55^ The quality and the region of the pain syndrome define the treatment. CRPS, intractable cancer pain, erythromelalgia, and trigeminal neuralgia predominantly exhibit neuropathic characteristics.^56^ Nociceptive pain is more commonly associated with conditions like dystonic pain and spastic pain.^5^ This differentiation aids in tailoring interventions to the specific pain mechanisms underlying each condition, improving treatment outcomes.

Neurostimulation reduces pain by interfering with the transmission of pain through tactile or electrical stimulation.^5^ Examples of neurostimulators include SCS, peripheral nerve stimulation (PNS), MCS, DBS, and ONS. In adults, SCS and PNS are recognized as effective management options for chronic pain.^57^ Intracranial stimulation, such as MCS, DBS, or ONS, may be pursued when less invasive methods have proven ineffective. MCS holds promise in treating central pain syndromes such as thalamic pain syndrome, while DBS may be employed for various nociceptive and neuropathic pain states.^58^

Originally, we intended to explore the use of neurosurgical procedures for chronic and end‐of‐life pain in children and create standardized treatment guidelines, similar to those developed in adults. However, the use of heterogeneous non‐standardized pain measures made it difficult to compare the efficacy of neurosurgical procedures across studies. Therefore, we determined efficacy using only quantitative measures of pain control, defining effectiveness as a ≥50% reduction in the numeric pain score. Here, we present a focused review describing the available evidence of the neurosurgical treatment of chronic pain for children, as a first step towards standardized pain guidelines.

### Neuropathic pain

Neuropathic pain, characterized by hyperalgesia, allodynia, and paresthesia, originates as a result of damage or dysfunction of the central or peripheral nervous system.^56^ Neuropathic pain typically responds well to neurostimulation.^56^ Overall, 58% of the papers treating neuropathic pain utilized neurostimulation, with the majority reporting good efficacy.^30^, ^31^, ^35^, ^36^, ^37^, ^40^, ^42^, ^43^, ^44^, ^45^, ^46^, ^47^, ^48^, ^49^, ^50^, ^51^, ^52^, ^53^ Only one study reported non‐efficacy utilizing neurostimulation.^37^ Graca and colleagues utilized DRG stimulation for CRPS‐I, reporting efficacy in 60% of patients (3/5) with a reduction in NRS score ranging from 50% to 100%, while the other two patients had no response at all.^37^ One additional case report utilized DRG stimulation for CRPS‐I and reported a 100% reduction in pain.^36^ SCS was most commonly employed (35.5%) with all but 1 manuscript reporting efficacy.^31^, ^43^, ^44^, ^45^, ^46^, ^47^, ^48^, ^49^, ^50^, ^51^, ^52^ DRG stimulation was rarer (9.7%) and may require further research to determine optimal patient selection.^35^, ^36^, ^37^ ONS and MCS were each used in only one case report, which reported subjective improvement in pain but did not quantify the effect.^40^, ^42^

Neurostimulation should be considered for the treatment of chronic pediatric pain of neuropathic origin. This treatment option is particularly appealing in these patients due to its reversibility and absence of permanent damage. Ablative techniques may precipitate neuropathic pain and therefore may not represent an optimal treatment.^5^ However, our study found that 12.9% of manuscripts treating neuropathic pain utilized ablative techniques and noted objective or subjective improvement.^16^, ^17^, ^33^, ^39^ Sensory complications were noted following these procedures in 40% of patients with resolution over 3 months. Appropriate ablative techniques can be considered for neuropathic pediatric pain, although the higher risk of neuropathic pain or lack of efficacy should be considered and may not be suitable for candidates with a long‐life expectancy.^59^

Intrathecal pumps were used in 32.3% of papers for neuropathic pain, with variable efficacy.^18^, ^19^, ^20^, ^22^, ^23^, ^24^, ^26^, ^27^, ^28^, ^31^ Two papers reported non‐efficacy.^22^, ^31^ Complications such as device difficulties (eg, pump size, catheter blocking, catheter dislodgement), medication effects (eg, nausea, vomiting, sedation, respiratory depression, gastrointestinal issues), and surgical complications (eg, wound healing, post‐dural puncture headache) were reported.^22^, ^31^ Intrathecal pumps can be used to improve drug delivery and were most utilized for cancer pain.

### Nociceptive pain

Nociceptive pain occurs in response to transduction by activated nociceptors and continues via transmission along pain tracts, modulation within the CNS, and is ultimately perceived. Nociceptors, classified by their response profiles, can be activated by mechanical, thermal, and chemical stimuli.

Neurostimulation was used in 40% of manuscripts (2/5) to treat nociceptive pain, one using DBS demonstrated efficacy, while the other using ONS worsened the pain until the device was explanted.^34^, ^41^ More research is needed to determine if neurostimulation is an effective treatment for nociceptive pediatric pain.

Ablative therapy was used in 40% of papers (2/5) to treat nociceptive pain; both demonstrated efficacy.^32^, ^38^ Stereotactic mesencephalotomy was used for refractory head and neck pain secondary to a cervical cord glioblastoma and demonstrated a reduction in VAS score from 10 to 0.^32^ In adults, intractable cancer pain of the head and neck is a primary indication for stereotactic mesencephalotomy; application in children is extremely uncommon. In this case, the patient was able to remain pain‐free and leave the hospital for 5 months following this procedure.^32^ DRG radiofrequency ablation was used for the treatment of spastic pain in 17 children with cerebral palsy with a reduction in average VAS score from 8.39 to 4.17.^38^ Although many papers using rhizotomy were identified for the treatment of hypertonia, demonstrating a significant reduction in nociceptive pain, they were not included in this study because their primary purpose was for muscle tone control.^60^, ^61^, ^62^ Ablative procedures are effective for nociceptive pain control in pediatric patients and should be considered for medically refractory cases.

An intrathecal bupivacaine pump was used in one case series for the treatment of rapidly progressive pain due to arachnoiditis ossificans secondary to chemotherapy.^25^ The patient experienced a metallic taste and loss of appetite, which resolved over time. Intrathecal pumps can be used in nociceptive pain, though side effects may occur.

### Mixed pain

Mixed pain is the result of overlapping primary pain generators and includes nociceptive and neuropathic characteristics. This chronic pain was the least commonly reported (10% of manuscripts).^14^, ^15^, ^21^, ^29^ Neurostimulation was not reported for the treatment of mixed pain. Two papers reported the use of cordotomy both for intractable cancer pain and reported immediate and significant improvement in their pain.^14^, ^15^ Malignant pain with a life expectancy of <2 years is the primary indication for cordotomy in adults.^5^ One paper used intrathecal pumps in 25 children for various indications including end‐stage malignancy, extensive abdominal surgery, and trauma, noting a substantial reduction in pain and elimination or reduction of the need for supplemental opioids.^21^ Finally, one paper reported the use of midline myelotomy and an intrathecal pump for the treatment of intractable cancer pain.^29^ They reported pain relief from the midline myelotomy for 2 months and then, unfortunately, a complete return of their pain and only a moderate reduction in pain following intrathecal pump insertion (VAS score 9–7).

Mixed pain is complex, and reports of treatment in pediatric patients are extremely rare. Both intrathecal pumps and ablative techniques demonstrate subjective efficacy; however, combining therapeutic modalities did not appear to increase efficacy. A combination of neurostimulation with ablative or intrathecal pumps may provide relief in refractory patients. More research is required to discern optimal treatment strategies.

### Considerations

It is important to acknowledge differing viewpoints within the pediatric chronic pain community regarding the use of interventional procedures. Some experts argue that these should be a last resort after all other conservative measures have been exhausted due to concerns about the potential risks and long‐term impacts on a child. Our findings suggest that neurosurgical interventions may offer significant relief in cases where chronic pain severely impacts the child's quality of life. The decision to proceed with any neurosurgical intervention should take into account the individual needs of the patient and involve interdisciplinary discussions with both the patient and their family. The timing to proceed with neurosurgical intervention varied significantly, ranging from 2 weeks to 5 years (see Table 1). Intrathecal pain pumps were typically the quickest intervention, with time to decision in as little as 2 weeks following presentation. By contrast, ablative and neuromodulatory techniques took longer (4 months‐5 years), perhaps due to the perceived invasiveness of these techniques. Several factors contributed to these delays, most notably the desire to exhaust all non‐interventional pain management strategies due to safety concerns and the desire to avoid procedural intervention. Additional delays were caused by patient‐specific factors, such as neutropenia or thrombocytopenia, which required medical stabilization before neurosurgical intervention could proceed. In some cases, lengthy delays were due to limited access to interventional specialists and extended referral times. Neurosurgical intervention should be integrated in discussions of pain management early in the treatment process. Many patients experienced significant pain resolution shortly after intervention, suggesting that earlier and improved access to neurosurgical procedures could lead to faster pain control, improved outcomes, and reduced psychiatric complications associated with prolonged pain.

### Limitations

There are several limitations to this study. Most of the manuscripts identified were case reports, which limits generalizability. The considerable heterogeneity among included studies in terms of design, patient demographics, and outcome measures limited the ability to perform meta‐analyses and necessitated a narrative synthesis. Quality assessment revealed variability in study quality, predominantly case reports, with a lack of high‐quality randomized controlled trials. Short follow‐up periods in many studies may underestimate long‐term treatment effects. Despite efforts to minimize bias, inherent biases may exist in the included studies, potentially influencing findings. Non‐standardized measures or no numerical measures of pain control limit the ability to compare efficacy across studies. While providing valuable insights, future research should address these limitations to better inform clinical practice regarding neurosurgical interventions for pediatric chronic pain management.

## CONCLUSIONS

Neurosurgical treatments are safe for chronic and end‐of‐life pain syndromes in children. Broad efficacy cannot be determined due to sparse literature and inadequately quantified pain responses. Neuropathic pain appears to be best treated with neurostimulation, nociceptive pain with ablative techniques and possibly neurostimulation techniques, and mixed pain with intrathecal pumps and/or ablative techniques. Guidelines for escalating chronic and end‐of‐life pain management in pediatric patients should be updated to include neurosurgical treatments and appropriate outcome scales. Focused research on appropriate patients, available neurosurgical therapies, and pediatric outcomes is warranted.

## AUTHOR CONTRIBUTIONS

SA contributed to study conception, completed data acquisition, analysis, and interpretation, and drafted the manuscript. NV completed data visualization and drafted the manuscript. GB completed data acquisition, analysis, and interpretation, and drafted the manuscript. JMM completed data analysis and revised the manuscript. SJA drafted the manuscript. KA and RDS critically revised the manuscript. JSR contributed to study conception, supervised the study, and revised the manuscript. All authors reviewed and approved the submitted manuscript.

## FUNDING INFORMATION

There was no specific funding received for the completion of this manuscript.

## CONFLICT OF INTEREST STATEMENT

Jeffrey S. Raskin is a paid consultant to Iota, Synergia, BlackRock Neurotech, and Medtronic. The other authors have no potential conflicts of interest to disclose.

## PATIENT CONSENT STATEMENT

Not applicable.

## PERMISSION TO REPRODUCE MATERIAL FROM OTHER SOURCES

Not applicable.

## CLINICAL TRIAL REGISTRATION

Not applicable.

## Supporting information


Data S1.

## Data Availability

All data supporting the findings of this study are available within this paper or supplementary materials.

## References

[1] Smith TJ , Hillner BE . The cost of pain. JAMA Netw Open. 2019;2(4):e191532. 10.1001/jamanetworkopen.2019.1532 30951152

[2] Tutelman PR , Langley CL , Chambers CT , Parker JA , Finley GA , Chapman D , et al. Epidemiology of chronic pain in children and adolescents: a protocol for a systematic review update. BMJ Open. 2021;11(2):e043675. 10.1136/bmjopen-2020-043675 PMC7888311 33593785

[3] Mathews L . Pain in children: neglected, unaddressed and mismanaged. Indian J Palliat Care. 2011;17(Suppl):S70–S73. 10.4103/0973-1075.76247 21811376 PMC3140088

[4] Odell S , Logan DE . Pediatric pain management: the multidisciplinary approach. J Pain Res. 2013;6:785–790. 10.2147/jpr.S37434 24250232 PMC3829620

[5] Sola RG , Pulido P . Neurosurgical treatment of pain. Brain Sci. 2022;12(11):1584. 10.3390/brainsci12111584 36421909 PMC9688870

[6] Anekar A , Hendrix J , Cascella M . WHO Analgesic Ladder. Tampa/St. Petersburg, FL: StatPearls Publishing; 2024. https://www.ncbi.nlm.nih.gov/books/NBK554435/

[7] Shah RD , Cappiello D , Suresh S . Interventional procedures for chronic pain in children and adolescents: a review of the current evidence. Pain Pract. 2016;16(3):359–369. 10.1111/papr.12285 25753547

[8] Walker SM . Pain in children: recent advances and ongoing challenges. Br J Anaesth. 2008;101(1):101–110. 10.1093/bja/aen097 18430745

[9] Kela I , Kakarala CL , Hassan M , Belavadi R , Gudigopuram SVR , Raguthu CC , et al. Chronic pain: a complex condition with a multi‐tangential approach. Cureus. 2021;13(11):e19850. 10.7759/cureus.19850 34963858 PMC8703086

[10] Page MJ , McKenzie JE , Bossuyt PM , Boutron I , Hoffmann TC , Mulrow CD , et al. The PRISMA 2020 statement: an updated guideline for reporting systematic reviews. BMJ. 2021;372:n71. 10.1136/bmj.n71 33782057 PMC8005924

[11] Bae G , Abdelmageed S . Neurosurgical Treatments for Pediatric Chronic Pain: A systematic review. York: PROSPERO; 2024. CRD42024521065. https://www.crd.york.ac.uk/prospero/display_record.php?ID=CRD42024521065

[12] Sterne JA , Hernán MA , Reeves BC , Savović J , Berkman ND , Viswanathan M , et al. ROBINS‐I: a tool for assessing risk of bias in non‐randomised studies of interventions. BMJ. 2016;355:i4919. 10.1136/bmj.i4919 27733354 PMC5062054

[13] Shlobin NA , Hoffman SC , Clark JR , Hopkins BS , Kesavabhotla K , Dahdaleh NS . Social media in neurosurgery: a systematic review. World Neurosurg. 2021;149:38–50. 10.1016/j.wneu.2021.01.135 33556595

[14] Reddy GD , Okhuysen‐Cawley R , Harsh V , Viswanathan A . Percutaneous CT‐guided cordotomy for the treatment of pediatric cancer pain. J Neurosurg Pediatr. 2013;12(1):93–96. 10.3171/2013.4.Peds12474 23682820

[15] Steel D , Kirkman MA , Thompson DNP , Aquilina K . Open thoracic anterolateral cordotomy for pain relief in children: report of 2 cases. J Neurosurg Pediatr. 2017;20(3):278–283. 10.3171/2017.5.Peds17119 28686123

[16] Kanpolat Y , Al‐Beyati E , Ugur HC , Akpinar G , Kahilogullari G , Bozkurt M . A curative treatment option for complex regional pain syndrome (CRPS) type I: dorsal root entry zone operation (report of two cases). Turk Neurosurg. 2014;24(1):127–130. 10.5137/1019-5149.Jtn.7997-13.0 24535809

[17] Iglesias B , Pérez Zabala J , Argañaraz R , Mantese B . Lumbosacral DREZotomy for oncologic pain treatment: a case‐based review. Childs Nerv Syst. 2023;39(1):41–45. 10.1007/s00381-022-05622-4 35970942

[18] D'Angelo R , Cohen IT , Brandom BW . Continuous epidural infusion of bupivacaine and fentanyl for erythromelalgia in an adolescent. Anesth Analg. 1992;74(1):142–144. 1734777

[19] Collins JJ , Grier HE , Sethna NF , Wilder RT , Berde CB . Regional anesthesia for pain associated with terminal pediatric malignancy. Pain. 1996;65(1):63–69. 10.1016/0304-3959(95)00193-x 8826491

[20] Galloway K , Staats PS , Bowers DC . Intrathecal analgesia for children with cancer via implanted infusion pumps. Med Pediatr Oncol. 2000;34(4):265–267. 10.1002/(sici)1096-911x(200004)34:4<265::aid-mpo8>3.0.co;2-1 10742065

[21] Aram L , Krane EJ , Kozloski LJ , Yaster M . Tunneled epidural catheters for prolonged analgesia in pediatric patients. Anesth Analg. 2001;92(6):1432–1438. 10.1097/00000539-200106000-00016 11375820

[22] Stanton‐Hicks M , Kapural L . An effective treatment of severe complex regional pain syndrome type 1 in a child using high doses of intrathecal ziconotide. J Pain Symptom Manage. 2006;32(6):509–511. 10.1016/j.jpainsymman.2006.08.002 17157748

[23] Farid IS , Heiner EJ . Intrathecal local anesthetic infusion as a treatment for complex regional pain syndrome in a child. Anesth Analg. 2007;104(5):1078–1180. 10.1213/01.ane.0000260563.39299.9c 17456655

[24] Kajiume T , Sera Y , Nakanuno R , Ogura T , Karakawa S , Kobayakawa M , et al. Continuous intravenous infusion of ketamine and lidocaine as adjuvant analgesics in a 5‐year‐old patient with neuropathic cancer pain. J Palliat Med. 2012;15(6):719–722. 10.1089/jpm.2011.0097 22401313

[25] Moens M , De Smedt A , Mariën P , Brouns R . Intrathecal bupivacaine for arachnoiditis ossificans: a case report. Clin Neurol Neurosurg. 2013;115(7):1162–1163. 10.1016/j.clineuro.2012.09.017 23040246

[26] Bengali R , Huang MS , Gulur P . The use of an intrathecal pump to manage intractable cancer pain in a pediatric patient: a case report. J Pediatr Hematol Oncol. 2014;36(3):e162–e164. 10.1097/MPH.0b013e31828e5dca 23652866

[27] Abolhasan Gharehdaghi F , Shahverdi E , Niktoreh Mofrad N , Faranoush M , Ebadi A . Does a single dose of adenosine in epidural space reduce cancer‐related neuropathic pain? A randomized clinical trial. Research Article. Int J Cancer Manag. 2018;11(5):e61872. 10.5812/ijcm.61872

[28] Tubic G . Epidural anesthesia to effectively manage pain and facilitate rehabilitation in a pediatric case of complex regional pain syndrome. Pain Manag Case Rep. 2018;2(6):209–212. 10.36076/pmcr.2018/2/209

[29] Bentley JN , Viswanathan A , Rosenberg WS , Patil PG . Treatment of medically refractory cancer pain with a combination of intrathecal neuromodulation and neurosurgical ablation: case series and literature review. Pain Med. 2014;15(9):1488–1495. 10.1111/pme.12481 24931480

[30] Rodriguez‐Lopez MJ , Fernandez‐Baena M , Barroso A , Yáñez‐Santos JA . Complex regional pain syndrome in children: a multidisciplinary approach and invasive techniques for the Management of Nonresponders. Pain Pract. 2015;15(8):e81–e89. 10.1111/papr.12317 26095620

[31] Kim E , Gamble S , Schwartz A , Cucchiaro G . Neuromodulation in pediatrics: case series. Clin J Pain. 2018;34(11):983–990. 10.1097/ajp.0000000000000632 29794496

[32] Ivanishvili Z , Pujara S , Honey CM , Chang S , Honey CR . Stereotactic mesencephalotomy for palliative care pain control: a case report, literature review and plea to rediscover this operation. Br J Neurosurg. 2016;30(4):444–447. 10.3109/02688697.2015.1133805 26760110

[33] Kato T , Sawamura Y , Abe H . Trigeminal neuralgia caused by a cerebellopontine‐angle lipoma: case report. Surg Neurol. 1995;44(1):33–35. 10.1016/0090-3019(95)00056-9 7482251

[34] Perides S , Lin JP , Lee G , Gimeno H , Lumsden DE , Ashkan K , et al. Deep brain stimulation reduces pain in children with dystonia, including in dyskinetic cerebral palsy. Dev Med Child Neurol. 2020;62(8):917–925. 10.1111/dmcn.14555 32386250

[35] Mol FMU , Roumen RMH . DRG spinal cord stimulation as solution for patients with severe pain due to anterior cutaneous nerve entrapment syndrome: a case series. Neuromodulation. 2018;21(3):317–319. 10.1111/ner.12692 28940994

[36] Pinckard‐Dover H , Palmer A , Petersen EA . A review of Neuromodulation for treatment of complex regional pain syndrome in pediatric patients and novel use of dorsal root ganglion stimulation in an adolescent patient with 30‐month follow‐up. Neuromodulation. 2021;24(4):634–638. 10.1111/ner.13257 32856364

[37] Graca MJ , Austell BT , Sremac AC , Lubenow TR . Dorsal root ganglion stimulation therapy in pediatric patients: a case series. Pain Med Case Rep. 2021;5(8):385–388. 10.36076/pmcr.2021.5.385

[38] Vles GF , Vles JS , van Kleef M , van Zundert J , Staal HM , Weber WE , et al. Percutaneous radiofrequency lesions adjacent to the dorsal root ganglion alleviate spasticity and pain in children with cerebral palsy: pilot study in 17 patients. BMC Neurol. 2010;10:52. 10.1186/1471-2377-10-52 20569438 PMC2909941

[39] Apiliogullari S , Aydin BK , Onal O , Kirac Y , Celik JB . Pulsed radiofrequency of dorsal root ganglia for the treatment of complex regional pain syndrome in an adolescent with poliomyelitis sequel: a case report. Pain Med. 2015;16(7):1369–1372. 10.1111/pme.12710 25688583

[40] Delavallee M , Rooijakkers H , Koerts G , Raftopoulos C . Motor cortex stimulation in a three‐year‐old child with trigeminal neuropathic pain caused by a malignant glioma in the cerebellopontine angle: case report. Neurosurgery. 2011;69(2):e494–e496. 10.1227/NEU.0b013e318218cf6f 21792145

[41] Stubberud A , Cheema S , Tronvik E , Matharu M . Nutcracker syndrome mimicking new daily persistent headache: a case report. Cephalalgia. 2020;40(9):1008–1011. 10.1177/0333102420918554 32295399 PMC7691626

[42] Borius PY , Valade D . A new treatment option for children with refractory chronic paroxysmal hemicranias: occipital nerve stimulation. Pediatr Neurol. 2021;125:18–19. 10.1016/j.pediatrneurol.2021.09.010 34624605

[43] Olsson GL , Meyerson BA , Linderoth B . Spinal cord stimulation in adolescents with complex regional pain syndrome type I (CRPS‐I). Eur J Pain. 2008;12(1):53–59. 10.1016/j.ejpain.2007.02.007 17889577

[44] Patel N , Chen E , Cucchiaro G . The complexity of pain management in patients with erythromelalgia. A A Case Rep. 2015;5(9):151–153. 10.1213/xaa.0000000000000201 26528699

[45] Dones I , Zanin L , Marongiu I , Levi V , Chiapparini L , Rizzi M . Severe pain and edema due to a widespread Lymphangioma: disappearance of symptoms and reduction of lesion with spinal cord stimulation. World Neurosurg. 2016;93:487. 10.1016/j.wneu.2016.06.129 27402439

[46] Tyagi R , Kloepping C , Shah S . Spinal cord stimulation for recurrent tethered cord syndrome in a pediatric patient: case report. J Neurosurg Pediatr. 2016;18(1):105–110. 10.3171/2015.12.Peds14645 26942269

[47] Fan X , Bu H , Wen Y , Ma L , Huang C , Xu F , et al. Spinal cord stimulation in the treatment of pediatric Erythromelalgia. World Neurosurg. 2020;142:388–390. 10.1016/j.wneu.2020.06.231 32652278

[48] Hale JE , Cheng J . Spinal cord stimulation for neurogenic thoracic outlet syndrome: a case report. A A Pract. 2020;14(6):e01194. 10.1213/xaa.0000000000001194 32224698

[49] Bakr SM , Knight JA , Shlobin NA , Budnick H , Desai V , Hill H , et al. Spinal cord stimulation for treatment of chronic neuropathic pain in adolescent patients: a single‐institution series, systematic review, and individual participant data meta‐analysis. Neurosurg Focus. 2022;53(4):e13. 10.3171/2022.7.Focus22330 36183181

[50] Schatmeyer BA , Dodin R , Kinsman M , Garcia D . Spinal cord stimulator for the treatment of central neuropathic pain secondary to cervical syringomyelia: illustrative case. J Neurosurg Case Lessons. 2022;4(6):CASE22226. 10.3171/case22226 36088568 PMC9706329

[51] Toriya V , Vissarionov S , Savina M , Baindurashvili A . Surgical treatment of a patient with erythromelalgia (Mitchell's syndrome) using invasive spinal cord stimulation: a clinical case. Pediatr Traumatol. 2022;10:197–205. 10.17816/PTORS108045

[52] Zuo L , Su A , Shi Y , Li N , Chen S , Yang X . Case report: spinal cord stimulation in the treatment of pediatric erythromelalgia. Front Neurol. 2023;14:1143241. 10.3389/fneur.2023.1143241 37273700 PMC10233004

[53] Delye H , Lagae L , Vermylen J , Nuttin B . Thalamic stimulation as a treatment for primary erythromelalgia: technical case report. Neurosurgery. 2005;57(4 Suppl):e404. 10.1227/01.neu.0000176703.27632.6d 16234658

[54] Sava S , Lebel AA , Leslie DS , Drosos A , Berde C , Becerra L , et al. Challenges of functional imaging research of pain in children. Mol Pain. 2009;5:30. 10.1186/1744-8069-5-30 19531255 PMC2702328

[55] Nijs J , Leysen L , Adriaenssens N , Aguilar Ferrándiz ME , Devoogdt N , Tassenoy A , et al. Pain following cancer treatment: guidelines for the clinical classification of predominant neuropathic, nociceptive and central sensitization pain. Acta Oncol. 2016;55(6):659–663. 10.3109/0284186x.2016.1167958 27142228

[56] Cavalli E , Mammana S , Nicoletti F , Bramanti P , Mazzon E . The neuropathic pain: an overview of the current treatment and future therapeutic approaches. Int J Immunopathol Pharmacol. 2019;33:2058738419838383. 10.1177/2058738419838383 30900486 PMC6431761

[57] Hofmeister M , Memedovich A , Brown S , Saini M , Dowsett LE , Lorenzetti DL , et al. Effectiveness of Neurostimulation Technologies for the Management of chronic pain: a systematic review. Neuromodulation. 2020;23(2):150–157. 10.1111/ner.13020 31310417

[58] Bittar RG , Kar‐Purkayastha I , Owen SL , Bear RE , Green A , Wang SY , et al. Deep brain stimulation for pain relief: a meta‐analysis. J Clin Neurosci. 2005;12(5):515–519. 10.1016/j.jocn.2004.10.005 15993077

[59] Loeser J . Introduction: ablative neurosurgical operations. In: Bonica J , editor. The Management of Pain. Amsterdam: Lea and Febiger; 1990. p. 2040–2043.

[60] Abdel Ghany WA , Nada M , Mahran MA , Aboud A , Mahran MG , Nasef MAA , et al. Combined anterior and posterior lumbar Rhizotomy for treatment of mixed dystonia and spasticity in children with cerebral palsy. Neurosurgery. 2016;79(3):336–344. 10.1227/neu.0000000000001271 27244465 PMC4974062

[61] Tedroff K , Hägglund G , Miller F . Long‐term effects of selective dorsal rhizotomy in children with cerebral palsy: a systematic review. Dev Med Child Neurol. 2020;62(5):554–562. 10.1111/dmcn.14320 31342516 PMC7187377

[62] Peck J , Urits I , Kassem H , Lee C , Robinson W , Cornett EM , et al. Interventional approaches to pain and spasticity related to cerebral palsy. Psychopharmacol Bull. 2020;50(4 Suppl 1):108–120. 33633421 10.64719/pb.4385 PMC7901135

