# Supplementary material for: Neurosurgical management for chronic and end‐of‐life pain in children: A systematic review
Source: Pain Pract. 2025 Apr 10;25(5):e70034. doi: 10.1111/papr.70034 (PMC11983359; doi:10.1111/papr.70034)
Supplement: Supplementary file 1 — Data S1. [file PAPR-25-0-s001.docx]

**Supplementary Table S1.** Search terms

| **Database** | **Terms** |
| --- | --- |
| PubMed    990 | ((((((((((((Pain[MeSH Terms]) OR (pain)) OR (pain management[MeSH Terms])) OR ("pain management*")) OR (chronic pain[MeSH Terms])) OR (pain clinic[MeSH Terms])) OR (palliative care[MeSH Terms])) OR (care, palliative[MeSH Terms])) OR (terminal care[MeSH Terms])) OR ("chronic pain")) OR ("pain clinic")) OR ("terminal care")) OR ("palliative care")  AND  ((((((((child[MeSH Terms]) OR (child*)) OR (adolescent[MeSH Terms])) OR  (adolescent*)) OR (teen*)) OR (youth*)) OR (infant*)) OR Infant [MeSH]) OR  (pediatric*) OR (pediatric[MeSH Terms])  AND   (((((((((((((((((((((((((((((((((((((rhizotomy[MeSH Terms]) OR (rhizotomy*)) OR (deep brain stimulation[MeSH Terms])) OR ("deep brain stimulation*")) OR (spinal cord stimulation[MeSH Terms])) OR ("spinal cord stimulation*")) OR (cordotomy[MeSH Terms])) OR (cordotomy*)) OR ("midline myelotomy*")) OR ("drez lesion*")) OR ("trigeminal nucleotomy*")) OR (mesencephalotomy*)) OR (cingulotomy*)) OR ("trigeminal tractotomy*")) OR ("motor cortex stimulation*")) OR ("occipital nerve stimulation*")) OR ("dorsal root ganglia* stimulation*")) OR ("intrathecal opioid pump*")) OR (spinal nerve root/surgery[MeSH Terms])) OR (trigeminal caudal nucleus/surgery[MeSH Terms])) OR (mesencephalon/surgery[MeSH Terms])) OR (gyrus cinguli/surgery[MeSH Terms])) OR (trigeminal neuralgia/surgery[MeSH Terms])) OR (trigeminal nerve/surgery[MeSH Terms])) OR (spinal ganglia/surgery[MeSH Terms])) OR (implantable infusion pump[MeSH Terms])) ) OR ("dorsal root entry zone lesion*")) OR (DBS)) OR (SCS)) OR ("DRG stimulation*") ) OR ("intrathecal lidocaine ")) OR ("intrathecal clonidine")) OR ("intrathecal morphine ")) OR ("intrathecal ziconotide ")) OR ("intrathecal hydromorphone")) OR ("intrathecal fentanyl")) OR ("intrathecal bupivacaine") |
| Scopus    915 | ( TITLE-ABS-KEY ( pain )  OR  TITLE-ABS-KEY ( "pain management*" )  OR  TITLE-ABS-KEY ( "chronic pain" )  OR  TITLE-ABS-KEY ( "pain clinic*" )  OR  TITLE-ABS-KEY ( "palliative care*" )  OR  TITLE-ABS-KEY ( "terminal care*" ) )  AND  ( TITLE-ABS-KEY ( child* )  OR  TITLE-ABS-KEY ( adolescent* )  OR  TITLE-ABS-KEY ( teen* )  OR  TITLE-ABS-KEY ( youth* )  OR  TITLE-ABS-KEY ( infant* )  OR  TITLE-ABS-KEY ( pediatric* ) )  AND  ( TITLE-ABS-KEY ( rhizotomy* )  OR  TITLE-ABS-KEY ( "deep brain stimulation" )  OR  TITLE-ABS-KEY ( "spinal cord stimulation" )  OR  TITLE-ABS-KEY ( cordotomy* )  OR  TITLE-ABS-KEY ( "midline myelotomy" )  OR  TITLE-ABS-KEY ( "drez lesion" )  OR  TITLE-ABS-KEY ( "trigeminal nucleotomy" )  OR  TITLE-ABS-KEY ( mesencephalotomy* )  OR  TITLE-ABS-KEY ( cingulotomy* )  OR  TITLE-ABS-KEY ( "trigeminal tractotomy" )  OR  TITLE-ABS-KEY ( "motor cortex stimulation" )  OR  TITLE-ABS-KEY ( "occipital nerve stimulation" )  OR  TITLE-ABS-KEY ( "dorsal root ganglia stimulation" )  OR  TITLE-ABS-KEY ( "intrathecal opioid pump" )  OR  TITLE-ABS-KEY ( "dorsal root entry zone lesion*" )  OR  TITLE-ABS-KEY ( dbs )  OR  TITLE-ABS-KEY ( scs )   OR  TITLE-ABS-KEY ( "DRG stimulation*" )  OR  TITLE-ABS-KEY ( "intrathecal lidocaine*" )  OR  TITLE-ABS-KEY ( "intrathecal clonidine*" )  OR  TITLE-ABS-KEY ( "intrathecal morphine*" )  OR  TITLE-ABS-KEY ( "intrathecal ziconotide*" )  OR  TITLE-ABS-KEY ( "intrathecal hydromorphone*" )  OR  TITLE-ABS-KEY ( "intrathecal fentanyl*" )  OR  TITLE-ABS-KEY ( "intrathecal bupivacaine *" ) ) |
| Embase  1,587 | (analgesia)/exp/mj OR ((pain)/exp) OR (pain management) OR ('chronic pain') OR (('pain clinic')/exp) OR (('palliative therapy')/exp) OR (('terminal care')/exp)  AND  ('child'/exp OR 'child'/exp/mj OR child OR adolescent* OR 'adolescent'/exp/mj OR adolescent OR teen* OR youth* OR infant* OR pediatric*) AND ('pediatrics'/exp/mj OR pediatrics)  AND  rhizotomy OR ('deep brain stimulation') OR ('spinal cord stimulation') OR ('cordotomy (spinal cord)') OR ('midline myelotomy') OR ('drez lesion') OR ('trigeminal nucleotomy') OR (mesencephalotomy) OR (cingulotomy) OR ('trigeminal tractotomy') OR ('motor cortex stimulation') OR ('occipital nerve stimulation') OR ('dorsal root ganglia stimulation') OR ('intrathecal opioid pump') OR ('dorsal root entry zone lesioning') OR ('dbs') OR ('scs') OR ('DRG stimulation') OR ('intrathecal lidocaine') OR ('intrathecal clonidine') OR ('intrathecal morphine') OR ('intrathecal ziconotide') OR ('intrathecal hydromorphone') OR (intrathecal fentanyl) OR ('intrathecal bupivacaine') |

Records identified from:

Databases (n = 3,492)

Other Sources (n = 0)

Records removed *before screening*:

Duplicate records removed

(n = 858)

Records marked as ineligible by automation tools (n = 0)

Records removed for other reasons (n = 0)

**Identification**

Articles screened by title and abstract

(n = 2,634)

Records excluded

(n = 2,495)

Full-text articles sought for retrieval

(n = 139)

Articles not retrieved

(n = 0)

**Screening**

Full-text articles excluded

(n = 99)

Full-text articles assessed for eligibility

(n = 139)

Articles included in review

(n = 40)

**Included**

**Supplementary Figure S1.** PRISMA flow diagram for systematic selection of articles
